# Supplementary material for: Collagen-Containing Fish Sidestream-Derived Protein Hydrolysates Support Skin Repair via Chemokine Induction
Source: Mar Drugs. 2021 Jul 15;19(7):396. doi: 10.3390/md19070396 (PMC8303758; doi:10.3390/md19070396)
Supplement: Supplementary file 1 [file marinedrugs-19-00396-s001.zip › marinedrugs-1279413-supplementary.pdf]

marine drugs

Supplementary Material for

Collagen-Containing Fish Sidestream-Derived Protein Hydrolysates Support Skin Repair via Chemokine Induction

Ioanna Lapi [1,2], Ourania Kolliniati [1,2], Tone Aspevik [3], Eleftherios E. Deiktakis [1,2], Konstantinos Axarlis [1,2], Maria G. Daskalaki [1,2], Eirini Dermitzaki [1,2], Maria Tzardi [4], Sotirios C. Kampranis [5], Zouhir El Marsni [6], Katerina C. Kousoulaki [3], Christos Tsatsanis [1,2,*] and Maria Venihaki [1,*]

1 Laboratory of Clinical Chemistry, Medical School, University of Crete, 70013 Heraklion, Greece; iwanna_lapi@hotmail.com (I.L.); raliakolliniatis21@gmail.com (O.K.); el.deiktakis@gmail.com (E.E.D.); mol-grad392@edu.biology.uoc.gr (K.A.); m.daskalaki@med.uoc.gr (M.G.D.); renaderm@med.uoc.gr (E.D.)
2 Institute of Molecular Biology and Biotechnology, Foundation for Research and Technology Hellas, 71100 Heraklion, Greece
3 Department of Nutrition and Feed Technology, Nofima AS, 5141 Bergen, Norway; tone.aspevik@nofima.no (T.A.); katerina.kousoulaki@nofima.no (K.C.K.)
4 Laboratory of Pathology, Medical School, University of Crete, 70013 Heraklion, Greece; tzardi@med.uoc.gr
5 Section of Plant Biochemistry, Department of Plant and Environmental Sciences, University of Copenhagen, Thorvaldsensvej 40, 1871 Frederiksberg, Denmark; soka@plen.ku.dk
6 Seagarden AS, Karmsund Fiskerihavn, Husøyvegen 278, 4262 Avaldsnes, Norway; zouhir.el.marsni@seagarden.no
* Correspondence: tsatsani@uoc.gr (C.T.); venihaki@med.uoc.gr (M.V.); Tel.: +30-2810394833 (C.T.); +30-2810394583 (M.V.)

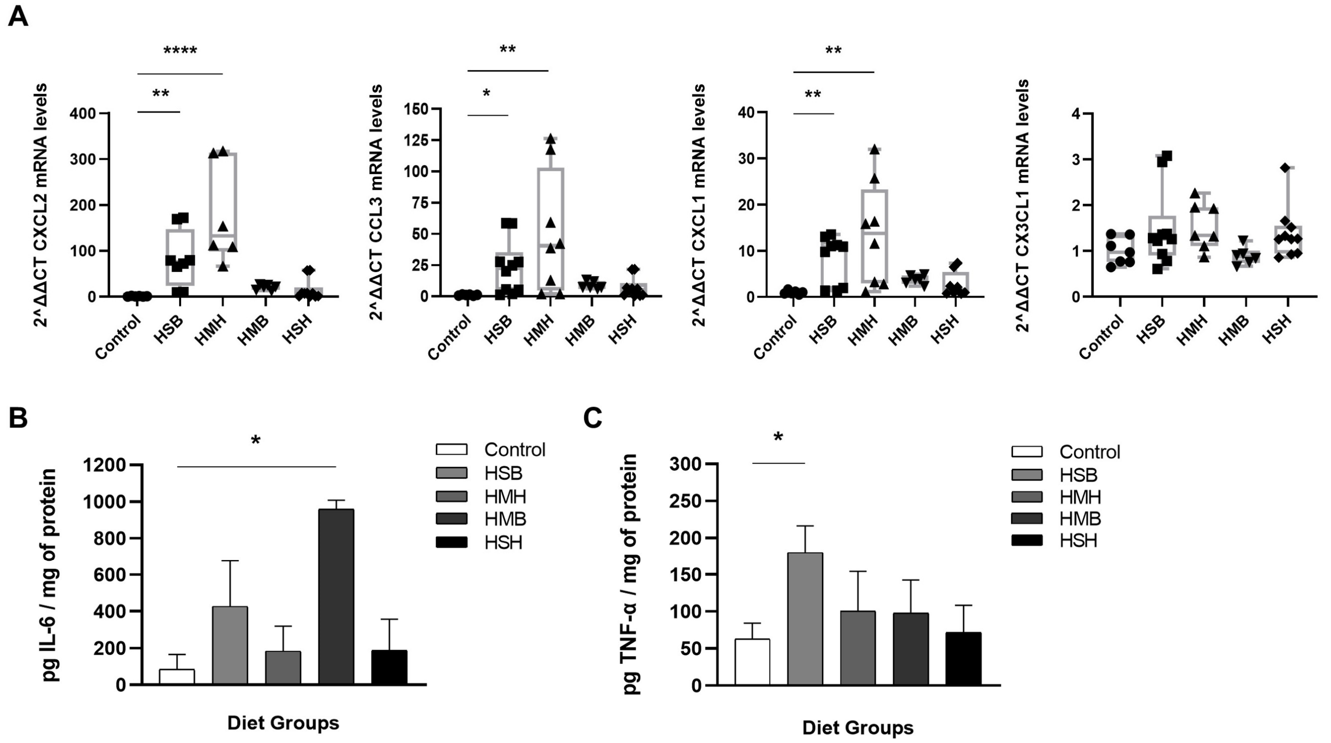

Supplementary Figure S1: The effect of different fish sidestream-derived extracts on chemokine expression. A. mRNA expression of CXCL2, CCL3, CXCL2 and CX3CL1 was analyzed by real-time PCR. IL-6 (B) and TNF-α (C) levels were quantified for each diet group via ELISA on the 5th day postinjury. *p<.005, **p<0.01, ***p<0.001, ****p<0.0001.

Supplementary Table S1. Chemical composition (g/100 g) of fish sidestream-derived supplements used in the cutaneous wound model.

Chemical composition	HMB	HMH	HSB	HSH	Collagen
Crude protein Kjeldahl (N x 6.25)	82.4	59.3	89.8	89.3	>90
Total dry matter	96.3	96.4	96.3	98	96.7
Ash	15.7	38.3	9.3	12.6	3.1
Water soluble crude protein	82	58.5	88.7	89.2	>95

Supplementary Table S2. Vitamin and mineral composition (mg/kg) of fish sidestream-derived supplements used in the cutaneous wound model.

Vitamins and minerals	HMB	HMH	HSB	HSH	Collagen
Salt (NaCl) (%)	8.6	3	3.8	8.6	0.6
Phosphorus (%)	1.1	0.37	0.82	0.48	0.02
Soluble phosphorus (%)	1.1	0.25	0.81	0.45	<0.1
Zink	9	5.5	7.4	65	0.8
Iron	6.5	7.2	3.2	4.9	1.3
Copper	17	13	3.8	5.6	1.8
Selenium	3.7	9.8	0.4	0.6	0.6
Manganese	0.2	0.2	0.1	0.3	1.1
Magnesium	2100	2700	850	740	750
Iodide	1.1	2.2	1.2	6.9	0.9
B1	1.65	9	3.11	4.2	<0.15
B2	10.2	21.2	6.57	6.49	1.03
B3	706	253	0.428	190	<1
B5	36.8	32.4	111	117	2.83
B6	<0.1	3.31	9.22	4.18	<0.1
B9	0.513	0.863	0.53	0.6	0.103
B12	0.374	0.387	0.177	0.268	0.0066

Supplementary Table S3. Peptide size distribution expressed in percentage of water-soluble peptides of fish sidestream-derived supplements used in the cutaneous wound model.

Molecular weight of peptide (Da)	HMB	HMH	HSB	HSH	Collagen
> 20000	<0.1	<0.1	<0.1	<0.1	<0.1
20000-15000	<0.1	<0.1	<0.1	<0.1	<0.1
15000-10000	0.1	0.1	0.1	0.1	0.1
10000-8000	0.1	0.3	0.2	0.2	0.5
8000-6000	0.5	1	0.9	1	2.4
6000-4000	1.9	3.7	3.3	3.6	9.4
4000-2000	8.2	13.9	12.9	15.9	28.1
2000-1000	14.5	17.7	18.4	22.2	28
1000-500	17.7	16.6	18.9	19	17.6
500-200	19.7	16.9	20.3	17.3	9.3
200-	37.3	29.7	24.9	20.7	4.6

Supplementary Table S4. Levels of total amino acids (g/100 g protein) of fish sidestream-derived supplements used in the cutaneous wound model.

Total amino acids	HMB	HMH	HSB	HSH	Collagen
Aspartic acid	6.5	4.4	7	6.8	6.4
Glutamic acid	10.7	7.4	10.9	10.8	10
Hydroxyproline	1.4	1.9	3	4.1	9.8
Serine	3.1	2.5	3.4	3.8	6.1
Glycine	5.3	6.2	8.8	12.1	26
Histidine	5	1.8	1.8	1.7	1.1
Arginine	4.5	3.7	5.3	5.6	8.9
Threonine	2.9	2	3.2	2.9	2.8
Alanine	4.6	4.2	5.6	6	9.8
Proline	3	3.1	4.5	6.1	11.7
Tyrosine	1.7	1.1	1.7	1.4	0.37
Valine	3.1	2	3.2	2.8	1.9
Methionine	1.9	1.4	2.3	2.4	2.4
Isoleucine	2.4	1.5	2.6	2.1	1.1
Leucine	5	3.2	5	4.3	2.4
Phenylalanine	1.9	1.5	2.3	2.3	2.1
Lysine	6.9	4.3	6.5	5.5	3.7

Supplementary Table S5. Levels of free amino acids (g/100 g protein) of fish sidestream-derived supplements used in the cutaneous wound model.

Free amino acids	HMB	HMH	HSB	HSH	Collagen
Aspartic acid	0.06	0.07	0.09	0.1	0
Glutamic acid	0.39	0.27	0.36	0.32	0.01
Hydroxyproline	0.01	0.01	0.02	0.03	0
Serine	0.09	0.12	0.12	0.16	0.02
Asparagine	0.02	0.01	0.02	0.02	0.01
Glycine	0.15	0.21	0.15	0.21	0.05
Glutamine	0.21	0.43	0.43	0.45	0
Histidine	3.2	0.81	0.22	0.2	0
Threonine	0.1	0.1	0.14	0.14	0
Alanine	0.29	0.27	0.44	0.4	0.03
Arginine	0.25	0.34	0.19	0.21	0.02
Proline	0.06	0.09	0.1	0.07	0
Tyrosine	0.18	0.26	0.18	0.14	0.02
Valine	0.1	0.14	0.23	0.19	0.01
Methionine	0.29	0.36	0.47	0.37	0
Cysteine	0	0	0	0	0
Isoleucine	0.08	0.13	0.24	0.19	0.01
Leucine	0.42	0.78	0.83	0.72	0.04
Phenylalanine	0.3	0.43	0.48	0.67	0.07
Tryptophane	0.07	0.07	0.12	0.1	0
Lysine	0.56	0.39	0.4	0.3	0
Creatinine	0.94	0.15	0.4	0.34	0.07
β-alanine	0	0	0.14	0.21	0
Taurine	0.85	1.4	0.46	1.3	0.03
4-aminobutanoic acid	0	0	0	0.01	0

Citrulline	0	0	0	0.01	0
Carnosine	0.06	0.03	0.08	0.03	0.01
Anserine	0.11	0.05	2.3	0.64	0
L-Ornithine	0.02	0.02	0.02	0.02	0
